# Intervention strategies for Parkinson’s disease: the role of exercise and mitochondria

**DOI:** 10.3389/fnagi.2025.1519672

**Published:** 2025-05-14

**Authors:** Ganggang Xu, Chunlian Ma, Yi Yang

**Affiliations:** ^1^Hubei Key Laboratory of Exercise Training and Monitoring, Department of Sports Medicine, Wuhan Sports University, Wuhan, China; ^2^Physical Fitness Monitoring and Chronic Disease Intervention Research Center, Wuhan Sports University, Wuhan, China

**Keywords:** Parkinson’s disease, exercise, dopamine, mitochondria, PINK1

## Abstract

Parkinson’s disease (PD), a progressive neurodegenerative disorder with complex pathogenic mechanisms, exhibiting rising prevalence alongside global population aging. Its pathological hallmarks include substantial loss of dopaminergic neurons in the substantia nigra pars compacta, leading to motor symptoms (e.g., bradykinesia, rigidity) and non-motor manifestations (e.g., cognitive impairment, sleep disorders). Accumulating evidence underscores mitochondrial dysfunction—encompassing reactive oxygen species (ROS) overproduction, defective mitophagy, and impaired biogenesis—as an important contributor to PD pathogenesis. Exercise, endorsed by leading medical and sports authorities as a non-pharmacological therapeutic strategy. While mitochondrial dysfunction impairs cellular energetics in PD patients, exercise can re-establish mitochondrial homeostasis through multiple pathways: stimulating neuroprotective exerkines, regulating mitochondrial ROS balance, modulating mitochondrial biogenesis and mitophagy, and enhancing brain-derived neurotrophic factor production. Many studies demonstrate that aerobic, resistance, and mind-body exercises demonstrably improve gait stability, postural control, and cognitive function in PD patients. However, standardized exercise prescriptions for PD prevention and treatment remain underutilized in clinical practice. This review synthesizes mitochondrial pathophysiology in PD progression, exercise-mediated regulatory mechanisms, and evidence-based exercise protocols, proposing accessible exercise regimens to support PD management. By integrating molecular insights with practical strategies, this work provides foundational evidence for utilizing exercise as a non-medical intervention against PD.

## 1 Introduction

Parkinson’s disease (PD), a chronic progressive neurological disorder, arises from degeneration of dopaminergic neurons in the substantia nigra pars compacta (SNpc). Over the past two decades, PD incidence has risen substantially, positioning it as the second most prevalent neurodegenerative disease in the world with the fastest-growing patient population among neurological disorders (GBD 2016 Parkinson’s Disease Collaborators, 2018). Global prevalence currently refers to approximately 8.5 million individuals (GBD 2017 Disease and Injury Incidence and Prevalence Collaborators, 2018), with projections suggesting this figure will surpass 10 million by 2030 (GBD 2016 Neurology Collaborators, 2019). The hallmark neuropathological features of PD are the extensive loss of dopaminergic neurons in the SNpc, with a massive decrease in dopamine (DA), a catecholamine-containing neurotransmitter, which leads to blockage of dopaminergic afferent nerves in the basal ganglia and striatum. Meanwhile, intraneuronal accumulation of misfolded α-synuclein (α-syn) aggregates forms Lewy bodies, which ultimately lead to dopaminergic neuronal death (Bourdenx et al., 2020). These pathological changes disrupt basal ganglia circuitry essential for motor control, manifesting clinically as bradykinesia, resting tremor, and rigidity (Armstrong and Okun, 2020). As disease progression occurs, patients develop progressive gait dysfunction and fine motor impairment, ultimately compromising activities of daily living. Mitochondrial dysfunction emerges as an important mechanistic contributor to PD pathogenesis, with evidence implicating respiratory chain defects, impaired mitophagy, and compromised biogenesis pathways. However, no systematic framework currently integrates mitochondrial pathophysiology with exercise-mediated neuroprotection across preclinical and clinical domains, hindering the translation of mechanistic insights into optimized rehabilitation protocols.

Current diagnostic challenges stem from the insidious onset and non-specific early symptoms of PD, necessitating reliance on clinical history and neurological examination rather than objective biomarkers (Berg et al., 2021). While dopamine replacement therapies provide symptomatic relief, their inability to modify disease progression and associated side effects significantly limits long-term utility. These therapeutic limitations, compounded by the disease’s multidimensional symptom burden, impose substantial functional impairments and socioeconomic costs (Crowley et al., 2019), and highlight the need for complementary strategies addressing PD’s multifactorial nature.

Exercise presents a compelling adjunctive intervention, demonstrating superior safety profiles and cost-effectiveness compared to medications for PD patients (Janssen Daalen et al., 2022). Epidemiological evidence suggests that increasing the amount of time spent on exercising may delay PD onset in at-risk populations and reduce disease prevalence (Alwardat et al., 2019). The potential role of exercise has garnered increasing interest. Mechanistically, exercise may enhance mitochondrial quality control through these key pathways: neuroprotective exerkines production, biogenesis potentiation, mitophagy optimization, and reactive oxygen species (ROS) scavenging.

In this review, we conduct a comprehensive systematic analysis of mitochondrial pathophysiology in PD, the effects of exercise on experimental PD animal models and human populations, and exercise-induced neuroprotection mediated through mitochondrial modulation. Our work emphasizes the critical need to differentiate between disease-modifying mechanisms and symptomatic improvements, while systematically identifying unresolved knowledge gaps in the field, particularly those pertaining to optimizing exercise prescription protocols for heterogeneous PD populations. Through this synthesis, we propose evidence-based exercise programs and recommendations for PD rehabilitation. Collectively, this review seeks to establish a theoretical foundation and practical reference for implementing exercise interventions in PD prevention and disease management and provides actionable thresholds for personalized PD rehabilitation.

## 2 Mechanisms of mitochondrial dysfunction in Parkinson’s disease

**FIGURE 1:**
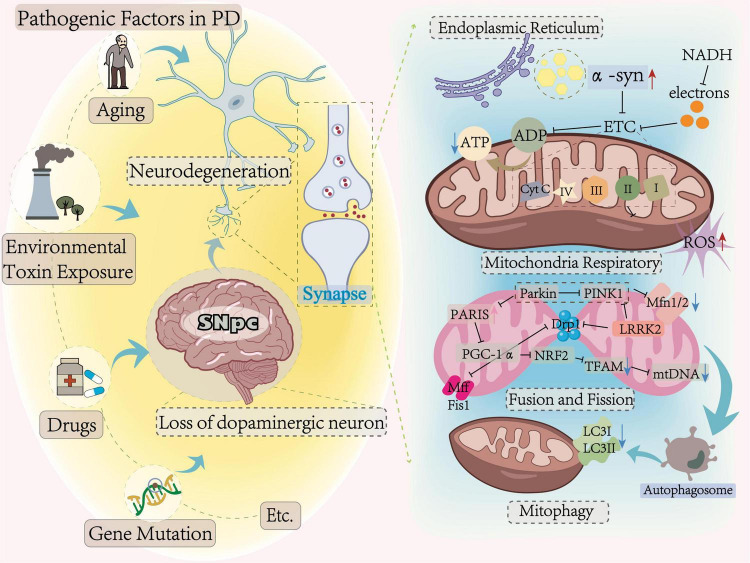
Parkinson’s disease-related mitochondrial dysfunction.

Mitochondria, the primary bioenergetic organelles in neurons, sustain cellular homeostasis through regulated oxidative phosphorylation—a process frequently disrupted in PD pathogenesis (Watts et al., 2018). Emerging evidence positions that mitochondrial dysregulation may contribute to PD pathology (Borsche et al., 2021; Simmons et al., 2020), alongside other mechanisms, such as neuroinflammation. In this chapter, we focus on key mitochondrial alterations, including ROS overproduction, and toxic α-syn aggregation, impaired mitochondrial biogenesis, and defective mitophagy (as shown in Figure 1). These processes may synergize with non-mitochondrial pathways to exacerbate neuronal vulnerability and neurodegeneration (Park et al., 2018).

### 2.1 ROS overproduction induces mitochondrial dysfunction

During mitochondrial oxidative phosphorylation, nicotinamide adenine dinucleotide hydrogen (NADH) from the tricarboxylic acid cycle is oxidized, providing electrons to the electron transport chain (ETC), with mitochondrial electron transport chain complex I (CoxI) serving as a key entry point for electrons. This process inherently generates superoxide anion O_2_^–^ (ROS), as metabolism byproduct (Kung et al., 2021). Notably, PD-related risk factors such as aging, drugs, and genetic mutations are associated with impair CoxI and CoxIII activities, potentially exacerbating ROS generation in dopaminergic neurons of SNpc (Del Rey et al., 2018). This dual pathology of elevated oxidative stress and compromised ATP synthesis may contribute to an imbalance between neuronal energy supply and demand (Trist et al., 2019). Pathological α-syn aggregates, which localize to mitochondria-associated membranes (MAMs), exert neurotoxic effects (Park et al., 2018), including reduced axon density, disrupted axonal transport, and inhibited dopamine release, collectively impairing synaptic transport function. Furthermore, α-syn oligomers interfere with CoxI-mediated respiration, induce selective oxidation of ATP synthase and peroxidation of mitochondrial lipids. Such cumulative damage could facilitate mitochondrial permeability transition pore opening, culminating in organelle swelling and irreversible cellular demise (Du et al., 2020).

### 2.2 Defective mitophagy induces mitochondrial dysfunction

Mitophagy, the selective removal of dysfunctional mitochondria, involves the engulfment of damaged mitochondria by autophagosomes, which subsequently fuse with lysosomes for degradation (Ganley, 2022). Studies have reported decreased levels of the autophagy marker microtubule-associated protein 1A/1B light chain 3I (LC3I)/LC3II were observed in MPP^+^ induced SNpc, suggesting potential dysregulation of autophagic flux in dopaminergic neurons (Lin et al., 2019). The PTEN-induced putative kinase 1 (PINK1)/Parkin is a key regulatory mechanism of mitophagy, where PINK1 stabilization on depolarized mitochondria and activates Parkin’s E3 ubiquitin ligase activity, this coordinated signaling initiates substrate ubiquitination for proteasomal degradation while recruiting autophagy machinery for mitochondrial clearance (Romaní-Aumedes et al., 2014; Yang et al., 2020). Gao et al. (2022) demonstrated that PINK1 knockout mice showed PINK1 deficiency is associated with synaptic dysfunction, attenuated long-term potentiation (LTP) associated with learning and memory, alongside reduced mitochondrial density and aberrant mitochondrial elongation. The pathophysiological cascade further involves leucine-rich repeat kinase 2 (LRRK2)-mediated disruption of PINK1/Parkin signaling. Mutant LRRK2 variants impede mitochondrial clearance through kinase-dependent interference with autophagosome recruitment, thereby creating a self-reinforcing cycle of organelle dysfunction (Korecka et al., 2019; Yang et al., 2020). Mitophagy impairment may also influence mitochondrial biogenesis via inhibiting peroxisome-proliferator-activated receptor γ coactivator-1α (PGC-1α) transcription (Piccinin et al., 2021). As the master regulator of mitochondrial biogenesis, PGC-1α coordinates nuclear respiratory factor 2 (NRF2)-mediated mitochondrial transcription factor A (TFAM) expression to drive mitochondrial DNA (mtDNA) replication and organelle proliferation (Lv et al., 2018). PD modeling with 6-hydroxydopamine (6-OHDA) -induced human neuroblastoma cell (SH-SY5Y) showed PGC-1α, NRF1, and TFAM expression levels were significantly reduced compared to controls, indicating impaired mitochondrial biogenesis (Lin C. et al., 2021). Similarly, 6-OHDA-induced PD mice showed reduced PGC-1α levels than control mice and reduced expression of mitochondrial fusin 1 (Mfn1) and Mfn2, which regulate mitochondrial fusion, suggesting PGC-1α deficiency may disrupt mitochondrial renewal through impaired fusion dynamics (Xi et al., 2018).

### 2.3 Impaired mitochondrial biogenesis induces mitochondrial dysfunction

Mitochondrial dynamics are linked to PD pathogenesis through potential imbalances in fission-fusion equilibrium. Dynamin-related protein 1 (Drp1), a key regulator of mitochondrial fission, has been reported to require LRRK2-mediated phosphorylation at Ser616 for proper membrane scission (Park et al., 2018). Under physiological conditions, Drp1 is recruited from the cytoplasm to the mitochondrial membrane and interacts with Drp1 receptors such as mitochondrial fission factor (Mff), mitochondrial fission protein 1 (Fis1). Drp1 and Drp2 oligomerize into ring-like structures around mitochondria, leading to mitochondrial membrane fission by guanosine triphosphatase (GTPase) hydrolysis (Simmons et al., 2020). Experimental studies show that PINK1 knockout mice exhibit cognitive impairments and synaptic dysfunction alongside reduced mitochondrial fission, while interventions enhancing Drp1 Ser616 phosphorylation appear to partially rescue these phenotypes (Gao et al., 2022). These observations suggest PINK1-mediated Drp1S616 phosphorylation may influence synaptic plasticity and mitochondrial dynamics. Other studies propose that mitochondria-localized ubiquitin ligase MITOL/March5 modulates mitochondrial dynamics by regulating Drp1, Fis1, mitochondrial fusion factor Mfn2, ubiquitination of fusion factors promotes mitochondrial network fragmentation, and accelerate the phagocytosis of mitochondria by autophagosomes (Barazzuol et al., 2020). Knockdown of MITOL in Hela cells significantly decreased the rate of Parkin recruitment, while its overexpression resulted in increased Parkin and TOM20 ubiquitination (Koyano et al., 2019). Therapeutic targeting of Drp1 phosphorylation demonstrates neuroprotective potential. Some reports indicate that inhibiting Drp1 dephosphorylation might block Drp1-mediated aberrant mitochondrial fission and significantly rescue part of motor function in PD mice (Zhang et al., 2019). Interestingly, others have found inhibition of Drp1 improves mitochondrial fission by upregulating PINK1/Parkin, increases in tyrosine hydroxylase (TH) expression in the substantia nigra and striatum, normalizes mitochondrial ultrastructure and fission function, ameliorates part of motor function in MPTP-induced PD mice (Feng et al., 2021). These divergent outcomes underscore the context-dependent nature of mitochondrial fission modulation, highlighting the need for further investigation into tissue-specific and disease-stage variations.

## 3 The effects of exercise exert on Parkinson’s disease

The Centers for Disease Control and Prevention (CDC) defines exercise as “any physical activity produced by the contraction of skeletal muscles that elevates energy expenditure above basal levels” and recognize exercise as a modulator of neurophysiological processes with implications for chronic disease prevention (Xu et al., 2019). Emerging evidence suggests that exercise improves muscle strength, balance and coordination and can be effective in preventing falls in older adults with PD (Sherrington et al., 2017). While studies have documented exercise-associated improvements in compensatory brain network function. Cognitive impairment, and neuroplasticity (Li et al., 2023), current evidence remains insufficient to establish definitive guidelines for exercise parameters (exercise type, intensity, duration) in neurodegenerative management. We categorized and compared the effects of different exercise types on PD patients (as shown in Table 1) with the aim of suggesting appropriate exercise programs for PD patients.

**TABLE 1:** The study on the effect of exercise in Parkinson’s disease (PD).

References	Study subjects	Exercise modality	Duration, frequency	Evaluated criteria	Significant outcomes
Dos et al., 2020	10 PD patients	Dance program	15 min for warm-up, stretching and body sense, 15 min for strength and balance, 15 min for movements exercise, 15 min for Rhythmic and playful exercises, 60 min per time, two times per week for 12 weeks	TUG^†^, gait kinematic analysis	A 12 weeks program of dance was sufficient to produce improvements in functional mobility and gait in patients with PD
Laat de et al., 2024	10 mild PD patients	High-intensity training and boxing	5 min warm-up, 30 min exercise (composed of strength, cardio, and power exercises), 15 min boxing, 10 min cool-down over 6 months	PET imaging, NM-MRI	Exercise reversed the expected decrease in DAT availability into a significant increase in both the substantia nigra and putamen, reversed the expected decrease in neuromelanin concentration in the substantia nigra into a significant increase
Johansson et al., 2022	26 PD patients	Cycling on a stationary bike	Three times per week for 30–45 min over 6 months	Resting-state functional, structural MRI	Aerobic exercise enhances functional connectivity between the anterior putamen and sensorimotor cortex, enhances functional connectivity of the right frontal-parietal network, improves cognitive control, reduces brain atrophy
van der Kolk et al., 2019	65 PD patients	Cycle on a stationary home-trainer	30–45 min (30 mins aerobic and 15 min warming up) at least three times per week for 6 months	MDS-UPDRS	Aerobic exercise attenuates motor symptoms in PD, improves cardiovascular fitness
Sacheli et al., 2019	20 idiopathic PD patients	Stationary cycling	30–50 min of cycling and 5–10 min of warm-up, three times per week for 3 months	MRI, [^11^C] raclopride positron emission tomography scans, motor and non-motor assessments	Aerobic exercise alters striatal responsiveness, promotes dopamine release in the caudate nucleus
Kanegusuku et al., 2017	15 PD patients	Resistance exercises program (horizontal leg press, squat, rotary calf, lateral pull down and chest press on isoinertial machines)	5 min warm-up and five resistance exercises, 2–4 sets, 6–12 repetitions maximum per set for 12 weeks	Spectral analysis of heart rate variability and cardiovascular responses to autonomic stress tests	Progressive resistance exercises improved cardiovascular autonomic dysfunction
Vieira de Moraes Filho et al., 2020	31 PD patients	resistance training (chest press, knee extension, hamstrings curl, leg press, seated row)	50–60 min with two sets of 10–12 repetitions, twice a week for 9 weeks	Bradykinesia UPDRS subscale, knee extensors isokinetic strength, 10 meters walk test, TUG, 30 s chair stand	Resistance training reduces bradykinesia and improves functional performance in patients with mild to moderate PD
Silva-Batista et al., 2020	17 PD patients	Individualized and adapted resistance training with instability	80–90 min each time, 3 days per week for 12 weeks	The new freezing of gait questionnaire, turning task, Stroop test, UPDRS, PD questionnaire, leg-lifting task, MRI	Adapted resistance training with instability can cause significant clinical improvement and brain plasticity in freezers
Li et al., 2024	143 PD patients	Tai Chi	Twice a week for 60 min each time over 3.5 year	UPDRS, TUG, the berg balance scale, the mini-mental state examination, the Parkinson’s Disease cognitive rating scale	Tai Chi has long-term beneficial effects on patients with PD, improving motor and non-motor symptoms and reducing complications
Yang et al., 2017	36 PD patients	Tai Chi	40–45 min each time, three times a week for 13 weeks	The non-motor symptoms scale, Parkinson’s disease sleep scale, Hamilton depression scale, Beijing version-Montreal cognitive assessment	Tai Chi improved patients’ global non-motor symptoms and sleep quality
Moon et al., 2019	30 PD patients	Qigong	15–20 min per time, Twice a day for 12 weeks	Clinical questionnaires and neuropsychological tests, inflammatory biomarker assays	Qigong has beneficial effects on non-motor symptoms and inflammatory status in patients with PD
Wang et al., 2020	23 elderly patients with mild to moderate PD	Wuqinxi	60 min per session, two sessions a week for 12 weeks	Purdue pegboard test, soda pop test, Parkinson’s disease questionnaire	Wuqinxi improved hand dexterity and motor function
Li et al., 2022	20 idiopathic PD patients	Wuqinxi	90 min per session, two sessions per week for 12 weeks	Gait parameters, MDS-UPDRS, mini-balance evaluation systems test, TUG, mini-mental state examination measured cognition, 39-item Parkinson’s disease questionnaire	Wuqinxi can improve patients’ gait, body movement flexibility, balance, improve their quality of life

^†^TUG, timed up and go test; PET imaging, positron emission tomography imaging; NM-MRI, neuromelanin-sensitive magnetic resonance imaging; DAT, dopamine transporter; MDS-UPDRS, the movement disorders society-unified Parkinson’s disease rating scale.

### 3.1 Aerobic exercise

Aerobic exercise refers to physical activities in which aerobic metabolism produces ATP for energy through oxidative phosphorylation, such as cycling, dancing, jogging, walking, can help improve executive function, cognitive function, motor skills, promote brain health (Bello et al., 2013; Robinson et al., 2019; Sharp and Hewitt, 2014). Compared to stretching and endurance exercise, aerobic exercise such as high-intensity interval training (HIIT) can delay the development of motor symptoms, improve quality of life and functional activity in PD patients to a greater extent (Ernst et al., 2024; Kathia et al., 2024). Among the many types of aerobic exercise, rhythmic aerobic exercise like dance is more effective in improving motor symptoms of PD patients (Cordani and Mosconi, 2024). Robinson et al. (2019) summarized treadmill exercise increased step length, stride length and pace, improved mobility in PD patients. Aerobic exercises such as running, aquatic obstacle training, or slackline, which focus on the lower extremities, are more suitable for improving the frozen gait caused by myotonia (Rutz and Benninger, 2020), and patients with severe motor symptoms of PD can perform deep water exercises, walking and rhythmic dance (Dos et al., 2020; Monteiro et al., 2017). However, it has also been suggested that aerobic exercise improves other symptoms of PD such as bradykinesia, myotonia and tremors to a lesser extent than other types of exercise (Lauzé et al., 2016). And current evidence regarding exercise-induced improvements in global cognitive function among PD patients remains limited. Further large-scale randomized controlled trials (RCTs) are required to confirm these findings and to identify the most effective type of exercise for cognitive enhancement (Folkerts et al., 2024).

### 3.2 Resistance exercise

Symptoms such as bradykinesia and muscle weakness highly affect the gait of PD patients and limit their range of motion, while resistance/strength exercise can improve the muscle strength of PD patients by enhancing muscle mass, bone density, and have a significant effect in improving bradykinesia (Palasz et al., 2019). After 9 weeks of resistance training in 25 PD patients, motor retardation subscale scores were reduced, knee extensor strength, walking ability, standing movements and other lower extremity mobility were improved (Vieira de Moraes Filho et al., 2020). Studies have shown that freezing gait was significantly improved in PD patients after 12 weeks of adaptive resistance training (Silva-Batista et al., 2020), with improved lower extremity muscle strength and quality of life (Yang and Wang, 2023). Resistance exercise with programmed increments in load intensity also promotes neuroplasticity in the brain, stimulates the secretion of neurotrophic factors, and enhances functional connectivity between different regions of the brain, providing cognitive benefits to PD patients (Chow et al., 2021). These findings position resistance exercise as a modulator of neuromuscular plasticity in PD.

### 3.3 Mind-body exercise

Mind-body exercises such as yoga, Tai Chi, Wuqinxi, Baduanjin are a combination of a series of body stretching exercises, spiritual meditation, respiration designed to promote physical and mental health and enhance physical fitness, and are widely used in rehabilitation exercises for PD patients because of their advantages of simple movements, fewer requirements for venues and equipment, low-intensity of exercises suitable for the elderly and patients with chronic diseases. Compared with resistance and stretching exercises, yoga may be more effective in relieving emotional problems such as anxiety and depression in PD patients (Kwok et al., 2023). Mind-body exercise, which emphasizes the dynamic modulation of posture and respiratory control, is considered to slow the progression of PD in patients, improve motor, balance, cognitive and respiratory functions (García-Muñoz et al., 2023), and enhance non-motor functions such as cognition, mood, and sleep (Yang et al., 2017). Long-term Tai Chi resulted in a significant reduction in the annual increments of levodopa equivalents in PD patients and showed that sustained mind-body exercise may alleviated the need for anti-PD medications (Li et al., 2024). Traditional Chinese fitness exercises Wuqinxi improved mood and significantly increased fine motor dexterity (Wang et al., 2020), while also enhancing motor symptoms, balance and quality of life in PD patients (Li et al., 2022).

### 3.4 Passive exercise

Severe neurodegenerative disorders induce progressive deterioration of gait and locomotor function through central and peripheral nervous system impairment, significantly compromising patients’ independence and quality of life. Given these constraints, passive exercise - encompassing both externally driven interventions (e.g., robot-assisted training) and whole-body vibration (WBV) therapy - has emerged as viable therapeutic strategies for PD patients with severe bradykinesia/rigidity who lack volitional movement capacity. In a randomized crossover trial involving 20 individuals with mild-to-moderate idiopathic PD, 30 min sessions of passive leg cycling at 60–80 revolutions per minute (RPM) significantly reduced tremor amplitude and improved bradykinesia scores (Ridgel et al., 2011). Several studies have demonstrated that WBV therapy, implemented through sustained maintenance of active or passive postures on mechanical vibration platforms, shows therapeutic potential in ameliorating motor symptoms among PD patients (Arenales Arauz et al., 2022; Dincher et al., 2019). Experimental findings demonstrate that a single session of WBV intervention elicits significant improvements in Timed Up and Go (TUG) test performance among PD patients, indicating enhanced neuromotor control of lower limb ambulatory function through proprioceptive pathway modulation (Sitjà Rabert et al., 2012).

### 3.5 Controversies perspectives on exercise interventions in PD

Current evidence regarding exercise efficacy in PD patients remains divergent perspectives. The primary Mendelian randomization analysis revealed no robust association between physical activity levels and PD risk (Wu et al., 2021), while resistance exercise exhibits variable functional outcomes across studies (de Almeida et al., 2025). Sangarapillai et al. (2021) reported no substantial improvements in gait parameters, including stride length and gait speed, following a 10-week boxing intervention. A meta-analysis further corroborates these observations, revealing no statistically significant effects of yoga on motor function improvement in PD patients. Another meta-analysis identified a non-linear dose- response relationship between exercise dosage and gait velocity improvement in PD patients (Xie et al., 2025). This temporal dissociation implies that sustained neuromodulation through movement-based interventions requires critical threshold accumulation.

Despite the well-documented benefits of exercise in PD, adherence to continuous exercise regimens remains challenging for many patients, primarily attributable to the interference of motor symptoms and the significant time required to complete the prescribed regimen. Additionally, safety concerns warrant careful consideration: unfit individuals engaged in (vigorous) exercise may increase the risk of sudden cardiac death and acute myocardial infarction (Franklin et al., 2020). Regarding the effects on adverse events, the most frequently reported events include falls and pain (Ernst et al., 2024). A cross-sectional web-based survey revealed that 45.1% of individuals with PD identified strenuous exercise/sports participation as fatigue-inducing factors (Lin C. et al., 2021). Another adverse event to consider is the risk of exercise-induced hypotension or post-exercise orthostatic hypotension (Low et al., 2014). Researchers highlighted that strenuous exercise protocols may induce substantial muscle damage, underlined the necessity for gradual implementation and close monitoring. Overall, the potential risks or adverse effects associated with exercise are relatively low in experimental settings, and these risks tend to diminish with prolonged exercise engagement over time, particularly when exercise modalities are matched to the individuals’ physical capabilities.

## 4 Potential mechanisms underlying exercise-induced neuroprotection in PD

### 4.1 Exercise produces neuroprotective exerkines

Exerkines constitute a diverse group of signaling molecules, encompassing simple organic acids, proteolytic enzymes, proteins, and microRNAs. These exercise-induced mediators are secreted by multiple tissues, including skeletal muscle, cardiac muscle, hepatic tissue, white adipose tissue (WAT), brown adipose tissue (BAT), and neuronal populations. Mitchell et al. (2024) highlighted the neuroprotective properties of exerkines, including anti-inflammatory, anti-apoptotic, and anti-oxidative mechanisms relevant to neurodegenerative pathophysiology. Pedersen et al. (2003) introduced the term “myokines” to describe muscle-secreted factors with autocrine, paracrine, and endocrine actions. Among these, Irisin is secreted in response to exercise stimulation, and demonstrates broad regulatory effects in optimizing autophagy, maintaining mitochondrial quality, alleviating oxidative stress and neuroinflammation, and regulating cell death-all processes intricately linked to the pathogenesis of PD (Qiu et al., 2024). Experiment reveals Irisin reduced motor deficits in α-syn preformed fibril mice model of sporadic PD, and inhibited the formation and propagation of α-syn (Kam et al., 2022). Mechanistic studies indicate that irisin restored part of mitochondrial populations in dopaminergic neurons of PD mice, concomitant with upregulated expression of SIRT1, PGC-1α, NRF-2, TFAM, and TOM20 (Zhang et al., 2023). Another myokine, Apelin-36, partially reverses dopamine depletion in MPTP-induced mice while enhancing antioxidant defenses (SOD and GSH) and reducing α-syn accumulation (Zhu et al., 2020). Similarly, the myokine FGF21 significantly increases TH expression in SNpc and striatum of PD mice, accompanied by elevated mitochondrial DNA copy numbers (Fang et al., 2020). All these studies show exercise-induced exerkines, particularly myokines, represent a promising therapeutic avenue for PD, modulating multiple pathological processes through their pleiotropic effects on mitochondrial function, oxidative stress, and neuronal survival.

### 4.2 Exercise improves mitochondrial respiration capacity

**FIGURE 2:**
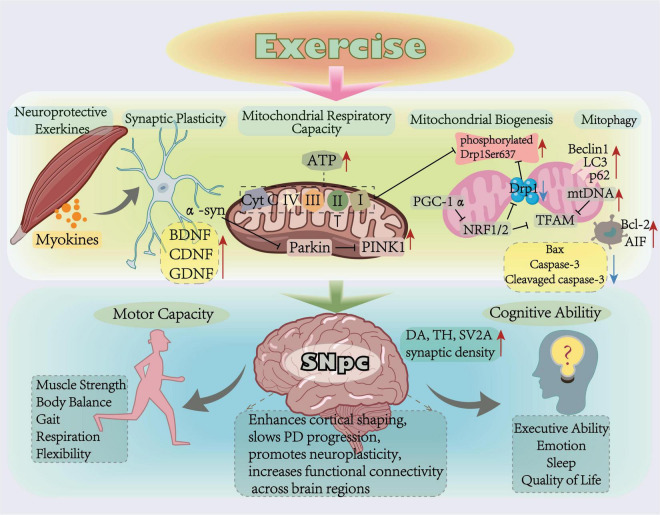
Mitochondrial related mechanism of exercise improving Parkinson’s disease.

Exercise improved gait deficits and limited dopaminergic neuron loss in PD rats, concomitant with elevated expression of TH and BDNF in the striatum and SNpc (Hsueh et al., 2018). The effects of exercise on neurological function are closely linked to mitochondrial alterations (as shown in Figure 2). Further studies on RNA extraction and sequencing of exercise-intervened PD mice have revealed that nigrostriatal neurotransmission influences dyskinesia, while the dorsolateral striatum that regulates movement is closely related to mitochondrial dysfunction (Klemann et al., 2018). When neuronal mitochondrial ETCs were disrupted with MPP^+^, mitochondrial damage increased and cell viability decreased. In contrast, TH expression was elevated in the SNpc of MPP^+^-induced PD mice following a 6 weeks treadmill exercise (12 months/min, 60 min/day, 5 days/week), along with restored mitochondrial CoxI-V expression, increased mitochondrial density, and elevated levels of phosphorylated Drp1Ser637, which was reduced by MPP^+^ treatment (Jang et al., 2018b). Similarly, 4 weeks of treadmill exercise (30 min/day at 15 months/min or 10 months/min, 40 min/day at 3 days/week) increased the expression of CoxI-V and TH, upregulated Parkin and PINK1 in the SNpc, decreased methamphetamine-induced rotations, and improved abnormal gait in PD rats (Chuang et al., 2017; Ferreira et al., 2020). CoxI is implicated in mitochondrial energy generation, these experimental evidence demonstrates that exercise mitigates energy production deficits by upregulating alternative electron transpoart pathways (e.g., CoxII-CoxV), thereby maintaining ATP synthesis and alleviating mitochondrial energy generation constraints in PD. PINK1 regulates mitochondrial respiration by affecting mitophagy, as described previously. Ebanks et al. (2021) performed gene enrichment analysis of PINK1 mutant *Drosophila* and found reduced expression of proteins related to mitochondrial respiration, oxidative phosphorylation, and energy metabolism. These protein profiles were restored to levels similar to wild-type *Drosophila* after 7 days of exercise (Ebanks et al., 2021). These experiments all suggest that exercise not only circumvent limitations imposed by reduced ATP efficiency, but also may improve dyskinesia caused by dopaminergic neuron degeneration through enhanced mitochondrial respiratory function.

### 4.3 Exercise promotes mitochondrial biogenesis

Exercise improves the brain’s ability to learn and memorize, which may be related to its role in promoting mitochondrial biogenesis. In healthy rats subjected to 12 weeks of continuous treadmill exercise (60 min/day, the velocity increased gradually from 18 months/min to 30 months/min), increased cortical CoxI and CoxV activity, elevated expression of PGC-1α and its transcriptional target TFAM, and reduced expression of the mitochondrial fission-associated protein Drp1 were observed. These findings indicate exercise may promote cortical mitochondrial biogenesis and autophagy (Marques-Aleixo et al., 2015). Rotenone, a potent inhibitor of CoxI causes ROS accumulation and neural mitochondrial damage, is commonly used for PD modeling. In PD rats induced by subcutaneous rotenone injection, locomotor activity was reduced, accompanied by gait abnormalities (shortened stride length, decreased step spacing, and increased overlap) and diminished striatal TFAM expression. However, after 30 min/day, five times/week for 4 weeks of treadmill exercise, these gait abnormalities were alleviated, and both TFAM and NRF-2 expression increased (Monir et al., 2020). Consistent with these findings, a recent study found that 4 weeks of treadmill exercise (10 months/min, 40 min/day, 3 days/week) significantly upregulated the expression of PGC-1α, NRF-1, and TFAM in the SNpc of PD rats compared to non-exercise controls (Ferreira et al., 2020). Collectively, these studies hint that exercise may exert dopaminergic neuroprotection by restoring mitochondrial biogenesis signaling and ameliorating motor symptoms in PD.

### 4.4 Exercise regulates mitophagy

Mitochondrial biogenesis is reduced due to intracellular α-syn accumulation, oxidative stress, and other neurodegenerative stressors. Exercise may protect cells by affecting mitophagy and demonstrate broad neuroprotective effects. A total of 8 weeks of treadmill exercise (10 months/min, 60 min/day, 5 days/week) ameliorated the MPTP-induced transport dysfunction in PD mice, decreased toxic α-syn levels, increased the expression of the anti-apoptotic protein B-cell lymphoma-2 (Bcl-2), and inhibited pro-apoptotic proteins Caspase-3, and Bcl-2-associated X (Bax) (Jang et al., 2018a). Rat subjected to hypoxia-ischemia exhibited motor and cognitive impairments concurrent with elevated expression of mitochondrial apoptosis—inducing proteins apoptosis inducing factor (AIF), cytochrome c, and cleaved Caspase-3 in brain tissues in the cytoplasm and nucleus of the cerebral cortex. After 4 weeks of swimming exercise, the expression of these proteins was reduced, and the rats’ motor, memory, and learning abilities improved (Gendi et al., 2022). This marked regulation of neuronal mitochondrial apoptosis by exercise enhances neuronal function. Exercise may increase mitophagy flux by activating the transcription of key autophagy genes, thereby enhancing mitophagy activity (Xing et al., 2019). After 12 months/min, 60 min/day, 5 days/week for 6 weeks of treadmill exercise, the expressions of LC3II, SQSTM1 (p62) and Beclin1 in healthy mice brains was significantly higher than in the non-exercise group. Further tests revealed elevated levels of the autophagy marker LC3 in mitochondria, proving that exercise may promote mitophagy (Kwon et al., 2021). Mitophagy deficits observed in neurodegenerative pathologies like Alzheimer’s disease are similarly mitigated by exercise: In APP/PS1 transgenic Alzheimer’s disease mice, 12 weeks of treadmill exercise (5 months/min for 5 min, 8 months/min for 5 min, 12 months/min for 30 min, and 5 months/min for 5 min, 45 min/day, 5 days/week) significantly increased hippocampal expression of the mitophagy-related proteins Parkin and LC3II and improved ultrastructure of mitochondria (Zhao et al., 2020). These findings collectively establish exercise-mediated mitophagy as a neuroprotective mechanism against mitochondrial dysfunction (Zhao et al., 2021).

### 4.5 Exercise improves synaptic plasticity

Although exercise cannot fully reverse neurodegeneration in the SNpc and striatum of PD, it alleviates motor and cognitive dysfunction by enhancing neurotransmitter and modulating neural plasticity (Guo et al., 2022; Petzinger et al., 2013). Aerobic exercise promotes structural and functional plasticity in the central nervous system, including synaptogenesis and angiogenesis (Cammisuli et al., 2020). Functional magnetic resonance imaging scans in PD patients post-exercise revealed increased ventral striatal activity and elevated dopamine release in the caudate nucleus (Sacheli et al., 2019). Studies show that aerobic exercise preserves dopaminergic networks integrity and enhances basal ganglia circuit plasticity (Palasz et al., 2019). After 12 months/min, 60 min/day, 5 days/week for 8 weeks of treadmill running in MPTP-induced PD mice, TH levels in the SNpc were increased, dopamine and BDNF levels in the hippocampus were increased, and synaptic ultrastructure was improved (Tang et al., 2023). Further investigations identified exercise-induced increases in synaptic vesicle glycoprotein 2A (SV2A) density within the thalamus and hippocampus of PD rats, indicative of enhanced synaptic vesicle number (Binda et al., 2021). Long-term exercise confers dopaminergic neuroprotection through sustained cerebral dopamine neurotrophic factor (CDNF) expression (Fallah Mohammadi et al., 2019). Additionally, exercise mitigates levodopa-induced dyskinesia (LID) and upregulates striatal glial cell-derived neurotrophic factor (GDNF) in PD mice (Speck et al., 2019).

## 5 Exercise programs and recommendations for patients with Parkinson’s disease

Clinically, neurodegenerative disease patients are encouraged to engage in early-stage rehabilitative protocols incorporating task-oriented functional exercises to restore motor deficits. While exercise-induced incremental effects remain small, these interventions retain therapeutic value in conferring functional benefits that improve quality of life and clinical outcomes. However, standardized exercise prescriptions remain elusive due to methodological heterogeneity across studies, encompassing exercise prescription parameters such as modality, frequency, intensity, duration, alongside interindividual variability in age, physical capacity, health status, cardiovascular risk profiles, and neurodegenerative symptom manifestations. Scholars suggest that maintaining at least 150 min of moderate or vigorous intensity exercise weekly mitigates PD risk (Lin et al., 2024). The metabolic equivalent of task (MET) is a unit used to calculate the intensity of exercise, and studies have concluded that maintaining an exercise intensity of 1,500 METs-min/week for aerobic exercise, 610 METs-min/week for resistance exercise, and 130–750 METs-min/week, can significantly improve motor function in PD patients (Yuan et al., 2024). A meta-analysis indicates that sustained multimodal interventions (> 850 METs-min/week for ≥ 18 weeks) yield significant motor symptom amelioration in PD patients (Gao et al., 2024). Based on relevant research, this review suggests that aerobic endurance and mind-body exercise should be used as the main form of exercise, supplemented by strength training to establish a population-wide exercise program for neurodegenerative disease prevention and management (as shown in Table 2). Regarding safety, vigorous exercise may transiently elevate cardiovascular risk and induce excessive fatigue in deconditioned individuals, thus researchers advocate for the adoption of a forced exercise paradigm. This approach facilitates augmented exercise intensity while circumventing disproportionate heart rate elevation and preventing undue fatigue (Miner et al., 2020).

**TABLE 2:** The exercise programs and recommendations for neurodegenerative disease.

Type of exercise	Frequency	Intensity	Time
Aerobic exercise (e.g., walking, running, cycling, swimming, dancing)	≥ 3 days/week	High-intensity (80%–85% HR_max_^‡^); moderate intensity (60%–65% HR_max_) (Yalçın Tavşan et al., 2024)	≥ 30 min accumulated exercise, 5 min warm-up and cool-down, progress to total of 150 min/week (Corcos et al., 2024)
Resistance exercise (patients should exercise the appropriate muscle groups accordingly)	≥ 2 days/week	30%–60% of 1RM for beginners and the elderly; 60%–80% of 1–4RM for advanced	1–4 sets of 8–15 repetitions, progress to 2–3 h/week (Martignon et al., 2021)
Mind-body exercise (e.g., yoga, Tai Chi) (Yang et al., 2017)	2–3 session/week	To the point of discomfort	45–60 min/session

^‡^HR_max_, maximum heart rate; 1RM, one-repetition maximum. If the patient experiences pain during exercise, stop exercising and seek medical help immediately. The exercise plan should be adjusted in time according to the individual’s physical fitness, disease stage and exercise experience. For patients with cognitive impairment, the exercise intensity should be appropriately reduced and a simple and easy-to-understand exercise pattern should be adopted. For patients with deficient motivation, physiotherapists and exercise professionals should enhance anticipatory rewards processing and utilize reinforcement strategies to improve adherence to exercise programs.

## 6 Limitations in the field and future perspectives for research

While current studies advance current understanding, the following aspects require prioritization to advance exercise and aging neuroscience: (1) The mechanisms linking exercise to mitochondrial quality control in neurodegeneration remain incompletely defined, particularly regarding the tripartite regulatory networks connecting exercise, mitophagy dynamics, and dopaminergic neuron survival. (2) Establishing population-specific exercise protocols demands a more systematic evaluation of dose-response relationships across PD subpopulations, optimizing exercise-medicated neuroprotective outcomes through type-, intensity-, and duration- specific parameterization. (3) Although the integration of multiple exercise modalities could potentially generate additive or synergistic therapeutic outcomes, this multimodal approach presents methodological limitations stemming from the inability to isolate and determine the specific contribution of individual exercise elements to the observed effects. (4) Long-term adherence patterns and sustainability of exercise-induced benefits in PD management remain understudied, requiring longitudinal investigations beyond controlled trial settings. Future research should prioritize large-scale, mechanistic studies across model systems to establish exercise as a scalable neuroprotective strategy.

## 7 Conclusion

The global burden of neurodegenerative disorders, exemplified by PD, continues to escalate, imposing substantial socioeconomic and healthcare burdens. Emerging evidence demonstrates that structured exercise interventions, particularly aerobic and mind-body exercise, can prevent and treat PD progression through risk mitigation in preclinical phases and motor function improvement in clinical populations. Meanwhile, with the development of new technologies, contemporary rehabilitation paradigms increasingly integrate wearable technologies and virtual reality (VR) systems, enabling precise exercise monitoring, task-oriented training, and remote supervision to optimize home-based intervention fidelity. Mechanistically, the neuroprotective efficacy of sustained aerobic exercise may correlate with multifaceted mitochondrial function, including mitochondrial respiratory function and energy metabolism, activation of biogenesis signaling cascades, promotion of mitophagy, and reduction of apoptosis. These coordinated adaptations collectively preserve dopaminergic neurotransmission capacity, thereby attenuating PD-associated functional decline.
